# SOX10 Transactivates S100B to Suppress Schwann Cell Proliferation and to Promote Myelination

**DOI:** 10.1371/journal.pone.0115400

**Published:** 2014-12-23

**Authors:** Sayaka Fujiwara, Shinya Hoshikawa, Takaaki Ueno, Makoto Hirata, Taku Saito, Toshiyuki Ikeda, Hiroshi Kawaguchi, Kozo Nakamura, Sakae Tanaka, Toru Ogata

**Affiliations:** 1 Departments of Sensory & Motor System Medicine, Faculty of Medicine, University of Tokyo, Tokyo, Japan; 2 Bone and Cartilage Regenerative Medicine, Faculty of Medicine, University of Tokyo, Tokyo, Japan; 3 Department of Rehabilitation for the Movement Functions, Research Institute, National Rehabilitation Center for Persons with Disabilities, Saitama, Japan; University of Oulu, Finland

## Abstract

Schwann cells are an important cell source for regenerative therapy for neural disorders. We investigated the role of the transcription factor sex determining region Y (SRY)-box 10 (SOX10) in the proliferation and myelination of Schwann cells. SOX10 is predominantly expressed in rat sciatic nerve-derived Schwann cells and is induced shortly after birth. Among transcription factors known to be important for the differentiation of Schwann cells, SOX10 potently transactivates the *S100B* promoter. In cultures of Schwann cells, overexpressing SOX10 dramatically induces S100B expression, while knocking down SOX10 with shRNA suppresses S100B expression. Here, we identify three core response elements of SOX10 in the *S100B* promoter and intron 1 with a putative SOX motif. Knockdown of either SOX10 or S100B enhances the proliferation of Schwann cells. In addition, using dissociated cultures of dorsal root ganglia, we demonstrate that suppressing S100B with shRNA impairs myelination of Schwann cells. These results suggest that the SOX10-S100B signaling axis critically regulates Schwann cell proliferation and myelination, and therefore is a putative therapeutic target for neuronal disorders.

## Introduction

Schwann cells have recently attracted great attention as a cell source for regenerative therapy for various kinds of neuronal disorders. Therefore, it is now crucial to elucidate the mechanisms of Schwann cell differentiation and function. Previous studies have clarified the role of various cytokines in Schwann cell proliferation and differentiation [1]. In addition, the developmental expression pattern of Schwann cell differentiation markers such as S100, nerve growth factor receptor (NGFR, also known as p75NTR), myelin associated glycoprotein (MAG), and myelin protein zero (MPZ, also known as P0), as well as transcription factors such as SOX10, paired box 3 (PAX3), POU class 3 homeobox 1 (POU3F, also known as Oct6), and early growth response 2 (EGR2, also known as KROX20) have been extensively studied [2], [3].

SOX family transcription factors are known to be involved in determining cell fate. Among the family members, SOX9 and SOX10 are involved in neural crest cell (NCC) migration and subsequently determining cell fate between neurons and Schwann cells [4]–[6]. Although the exact role of SOX10 in Schwann cell development still remains elusive, SOX10 is expressed from the early NCC stage through all stages of Schwann cell development and into adulthood [7].

S100 family proteins are abundantly expressed in glial cells, and some of the family members are implicated in a variety of intracellular and extracellular functions [8]. In the central nervous system (CNS), S100B promotes proliferation and inhibits differentiation of astrocytes [9], and increases in S100B are associated with neural diseases such as amyotrophic lateral sclerosis, multiple sclerosis, depression, Alzheimer's disease, and schizophrenia [10]–[13]. In addition, patients with Down's syndrome caused by chromosome 21 trisomy exhibit excessive expression of S100B, whose gene coding region is located on chromosome 21 [12], [14]. The expression of S100B gradually increases during Schwann cell differentiation [15], [16], and we previously reported that S100B expression is induced by SOX9 in chondrocytes [17]. Some studies point to an association between SOX10 and S100B; for instance, knockdown of SOX10 in Schwannoma cells drastically reduces S100B levels [18]. Waardenburg-Shah syndrome type 4, in which SOX10 mutations are observed, causes myelination disorders and peripheral neuropathy [19], [20]. Hirschsprung disease, also characterized by SOX10 mutations, causes the absence of the myenteric plexus where S100B is usually expressed [21]. In the present study, we identify S100B as one of the transcriptional targets of SOX10 during the differentiation of Schwann cells. We further found that the SOX10-S100B signaling axis regulates the proliferation and myelination of Schwann cells.

## Materials and Methods

### Cell cultures

All mouse experiments were performed according to the protocol approved by the Animal Care and Use Committee of the University of Tokyo. Carbon dioxide and decapitation were applied to euthanize adult and embryo rats, respectively. Primary rat Schwann cells were isolated and cultured as previously reported [22]. Briefly, we harvested Schwann cells from sciatic nerves of Wistar rats at postnatal day 2 (P2) and cultured the cells in DMEM containing 10% FBS and we added 10 µM AraC to the medium on the next day to eliminate contamination from fibroblasts. After 48 h, we replaced the medium with DMEM containing 3% FBS with 3 µM forskolin and 20 ng/mL neuregulin to expand the cells. We subcultured the cells by re-plating them onto poly-L-lysine-coated plastic dishes before confluence. We used Schwann cells between passages 3 and 7 in all experiments. For retrovirus infection, cells were incubated with DMEM containing retroviral vectors for 2 h, and then the medium was changed to DMEM with 10% FBS and cultured for an additional 48 h. Primary chondrocytes were isolated from the ribs of mouse embryos and cultured in DMEM with 10% FBS as previously described [23].

### Luciferase reporter assay

We cloned rat *S100b* promoter region from −1,000 to +200 bp relative to the transcriptional start site into the pGL3-Basic vector (Promega). We created deletion and mutation constructs by PCR. We cultured Schwann cells in medium containing 2 mM forskolin and 2 nM recombinant neuregulin (rh-HRG-1; Genzyme) before the transfection with the constructs and in medium with 2 mM forskolin and without neuregulin after the transfection. We performed luciferase assays with the PicaGene Dual SeaPansy Luminescence Kit (Toyo Ink) and present data as the ratio of the firefly activity to the Renilla activity.

### Chromatin immunoprecipitation (ChIP) assays

We performed ChIP assays in primary rat Schwann cells with an EZ ChIP kit (Upstate) as described by the manufacturer. Briefly, we obtained DNA, crosslinked to protein with formaldehyde, and sheared the DNA by pulsed ultra-sonication. For immunoprecipitation, we used an anti-SOX10 antibody (Santa Cruz) or a rabbit IgG antibody (negative control). Primer sets, spanning the identified response element, ranged from −421 to −157 bp (site AB), from −421 to −224 bp (site A), from −285 to −46 bp (site B), and from −78 to +169 bp (site D) relative to the transcriptional start site.

### Plasmids and viral vectors

We prepared shRNA vectors for rat *S100b*, rat *Sox10*, *GFP* and *luciferase* in piGENE mU6 vectors (iGENE Therapeutics). The sequence information of oligonucleotides synthesized for RNAi is available upon request. We subcloned the mouse U6 gene promoter and shRNA sequence from piGENE mU6 vectors into pMX–puro vectors to generate retroviral vectors as previously described [24].

### Real-time RT-PCR

We isolated total RNA from primary rat Schwann cells using Isogen (Wako) and an RNeasy Mini Kit (Qiagen). We used 1 µg of total RNA for a reverse transcription reaction with the QuantiTect Reverse Transcription kit (Qiagen) to generate single-stranded cDNA. We performed real-time PCR assays based on SYBR Green detection with the ABI Prism 7000 Sequence Detection system (Applied Biosystems). Copy numbers of target gene mRNA in each total RNA were calculated by reference to standard curves and were adjusted to the standard total RNA (Applied Biosystems), using beta-actin for normalization [17], [25]. We observed that the Ct values of the standard vector samples of each gene were similar to each other. All reactions were run in triplicate. Primer sequence information is available upon request.

### Western blotting

We lysed rat Schwann cells in M-Per mammalian protein extraction reagent (Pierce) containing a complete mini protease inhibitor cocktail tablet (Roche). For immunoblot analysis, lysates were fractionated by SDS-PAGE and transferred onto PVDF membranes (BIO-RAD). The membranes were incubated with an antibody to S100B (SH-B1, Sigma-Aldrich), SOX10 (N-20, Santa Cruz), or β-actin (Sigma-Aldrich). The immunoblots were developed by using the ECL system (Amersham Biosciences).

### Cell proliferation assay

We seeded Schwann cells at 10^3^ cells per well in a 96-well plate and evaluated cell proliferation by the modified MTT method designated WST-8 as indicated in the manufacture's instruction (Dojindo Laboratories) at the indicated time point. The absorbance of the product at the 490-nm wavelength was measured using an automated microplate reader (Bio-Tek Instruments). For BrdU detection analysis, we labeled the Schwann cells with 10 µM BrdU (Sigma) for 18 h and the cells were stained using a BrdU Immunohistochemistry System (Calbiochem). The cells were counterstained with the nuclear stain DAPI. The percentage of cells that had incorporated BrdU was quantified by determining the ratio of BrdU-positive nuclei and the total number of nuclei in 15 systematically sampled microscope fields.

### Myelination assay

We used dorsal root ganglion (DRG) neuronal coculture with Schwann cells as previously described [26]. Briefly, we isolated DRGs from embryonic rats at embryonic day (E) 15, dissociated with trypsin, and seeded them on 12-mm dishes coated with collagen type I at 200,000 cells per well. Non-neuronal cells were removed by treating cultures with C media (MEM, 10% FBS, 0.4% glucose, and 100 ng/mL 2.5S NGF) supplemented with 5-fluorodeoxyuridine, uridine, and AraC (10 µM) or with C media alone every 2–3 days alternately for 10 days. For myelination, 250,000 retrovirally-infected Schwann cells were seeded onto DRG cultures in DMEM/F12 medium with N2 supplement (Gibco) and 50 ng/mL NGF and kept for several days until Schwann cells populated axons. The medium was then switched to C media with 50 ng/mL ascorbic acid to promote myelination. Cocultures were kept for 2 weeks and then fixed with 4% paraformaldehyde. Anti-myelin basic protein (MBP) and anti-Tuj1 antibodies diluted at 1∶200 in TBST with 3% BSA were applied overnight at room temperature. Fluorescently-labeled secondary antibodies, Alexa Fluor 488 or 568, diluted at 1∶500 in TBST were then added to observe myelin-forming Schwann cells with fluorescence microscopy. The number of myelin-forming Schwann cells was counted by surveying 20 fields at 200× magnification from three independent experiments and expressed as a percentage ratio of control cultures.

### Statistical analysis

Group means were compared by ANOVA, and significance of differences were determined by post-hoc testing using Bonferroni's method.

## Results

### Expression of *Sox10* and *S100b* during Schwann cell differentiation

**Figure 1:**
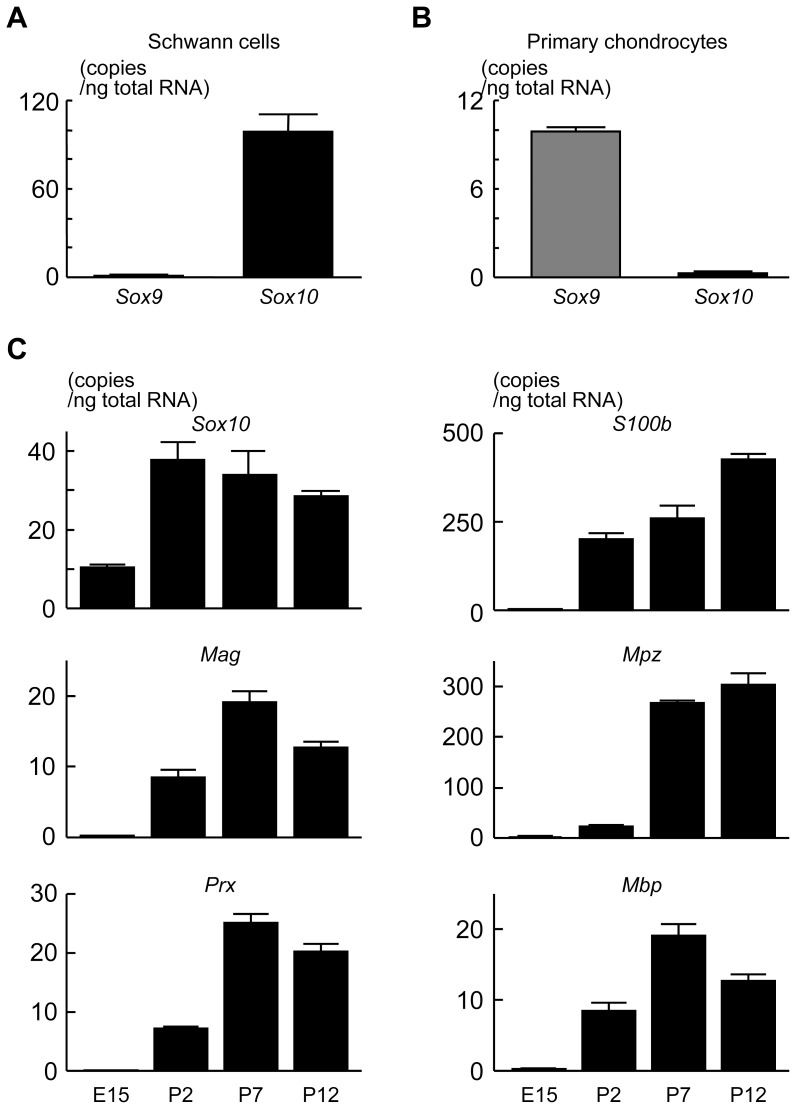
Expression pattern of *Sox10* and other differentiation markers in Schwann cells. (A and B) Comparison of expression level between *Sox10* and *Sox9* in primary rat sciatic nerve Schwann cells and primary rat rib chondrocytes. (C) Time course of mRNA levels of Schwann cell differentiation markers determined by real-time RT-PCR analysis during rat perinatal stages. All experiments were repeated independently three times with data shown as the mean (bars) ± SEM (error bars).

As shown in Fig. 1, *Sox10* was predominantly expressed in primary rat Schwann cells as compared to *Sox9* (Fig. 1A), while *Sox9* was highly expressed in primary rat rib chondrocytes as compared to *Sox10* (Fig. 1B). We then analyzed the expression of *Sox10* and other factors known to be involved in Schwann cell development in rat DRG at E15 and rat sciatic nerve at 2, 7, and 12 days after birth. *Sox10* expression markedly increased after birth and gradually decreased thereafter (Fig. 1C). The expressions of *S100b*, *Mpz*, *Mag*, *Periaxin* (*Prx*), and *Mbp* were induced after birth following *Sox10* induction (Fig. 1C).

### Regulation of S100B by SOX10

**Figure 2:**
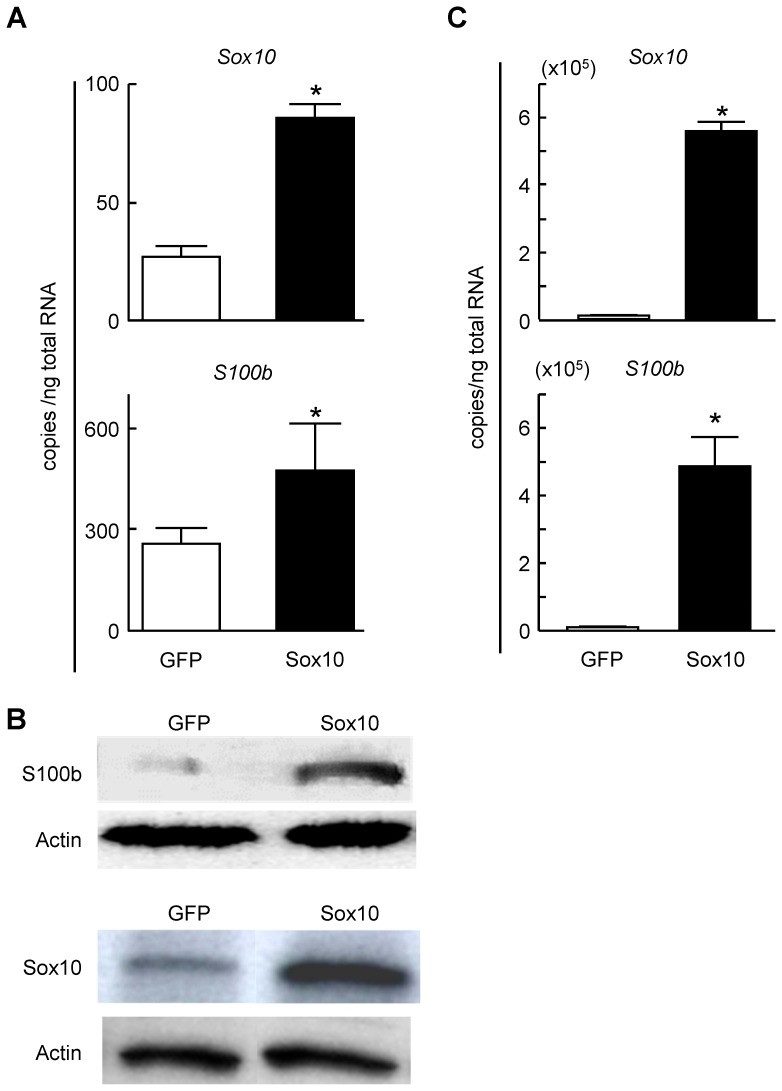
Modulation of S100B expression by SOX10 in Schwann cells. (A) mRNA levels of *Sox10* (top) and *S100b* (bottom) in stable lines of primary rat Schwann cells retrovirally transfected with SOX10 or control GFP. (B) Protein level of S100B and Sox10 in stable lines of primary rat Schwann cells retrovirally transfected with SOX10 or control GFP. (C) Modulation of *S100b* expression by SOX10 in ROS cells. mRNA levels of *Sox10* (top) and *S100b* (bottom) in stable lines of rat non-neurogenic ROS cells retrovirally transfected with SOX10 or control GFP. Experiments were repeated independently three times with data shown as the mean ± SEM. *P<0.05 versus control.

**Figure 3:**
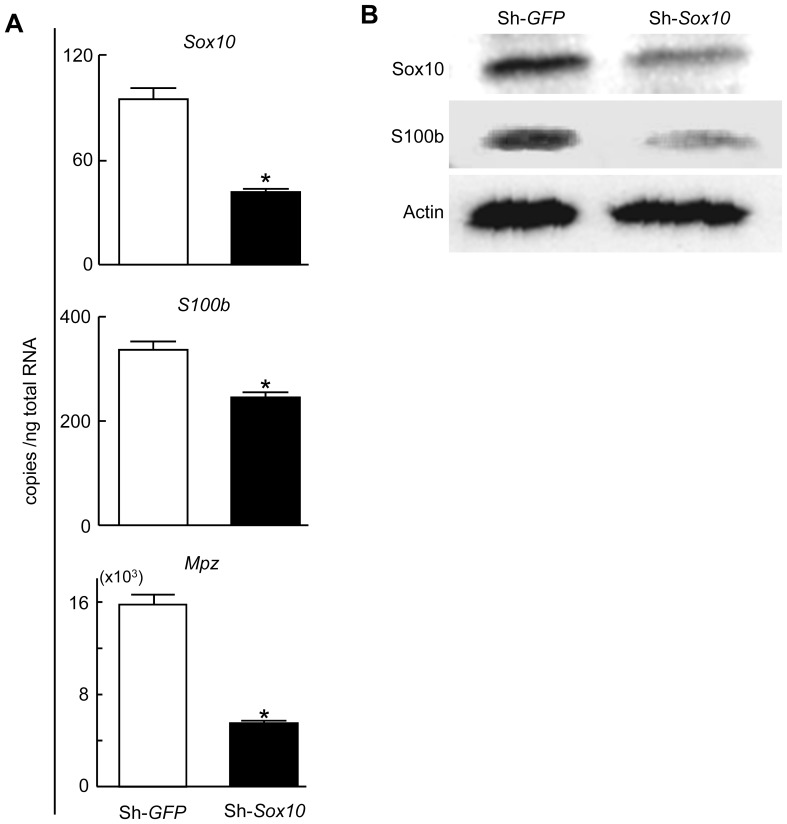
Suppression of S100B and Mpz expression by SOX10 insufficiency in Schwann cells. (A) mRNA levels of *Sox10* (top), *S100b* (middle) and *Mpz* (bottom) in stable lines of primary rat Schwann cells retrovirally transfected with shRNA specific for *Sox10* or *GFP*. All experiments were repeated independently three times with data shown as the mean ± SEM. *P<0.05 versus *GFP* or sh-*GFP*. (B) Protein levels of SOX10 and S100B in stable lines of primary rat Schwann cells retrovirally transfected with shRNA specific for SOX10 or control GFP.

To investigate the regulation of S100B by SOX10 in Schwann cells, we retrovirally overexpressed SOX10 in primary rat sciatic Schwann cells and rat osteosarcoma ROS cells (Fig. 2A, top). The expression level of S100B was significantly increased in SOX10-overexpressing Schwann cells (Fig. 2A, bottom and B), as well as in SOX10-overexpressing ROS cells (Fig. 2C). Conversely, when we suppressed SOX10 expression with a specific shRNA, the expression of S100B and Mpz was significantly suppressed (Fig. 3). These results suggest that SOX10 regulates S100B expression in Schwann cells.

**Figure 4:**
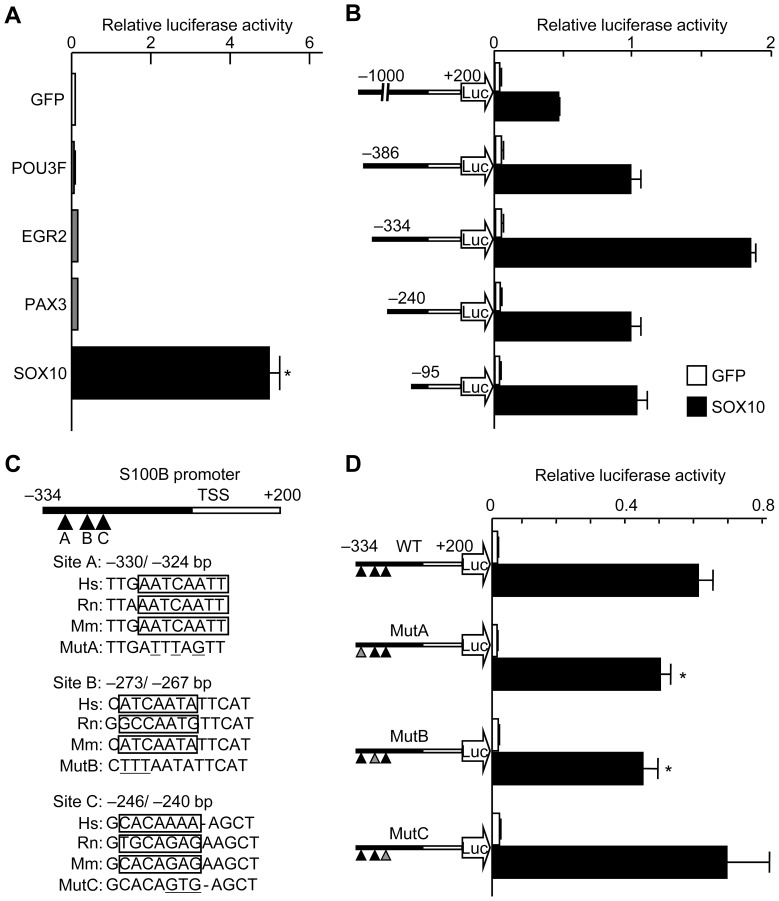
Identification of putative SOX10-response elements in S100B. (A) Luciferase activities after transfection of putative Schwann cell-related transcription factors into HeLa cells with a reporter construct containing a fragment (−1,000 to +200 bp) of the *S100B* gene. *P<0.05 versus GFP. (B) Deletion analysis using luciferase-reporter constructs containing a series of deletion fragments of the *S100B* gene in HeLa cells transfected with SOX10 or control GFP. (C) Comparison of human (Hs), rat (Rn), and mouse (Mm) sequences in three putative SOX motifs in the S100B promoter and mutated sequences (Mut A, Mut B, and Mut C), used in the following mutagenesis analysis. (D) Site-directed mutagenesis analysis using luciferase-reporter constructs containing −334 to +200 bp of the *S100B* gene with mutations as in Fig. 3C within the three SOX motifs in the cells above. *P<0.05 versus wild-type (WT) with SOX10. All experiments were repeated independently three times with data shown as the mean ± SEM.

**Figure 5:**
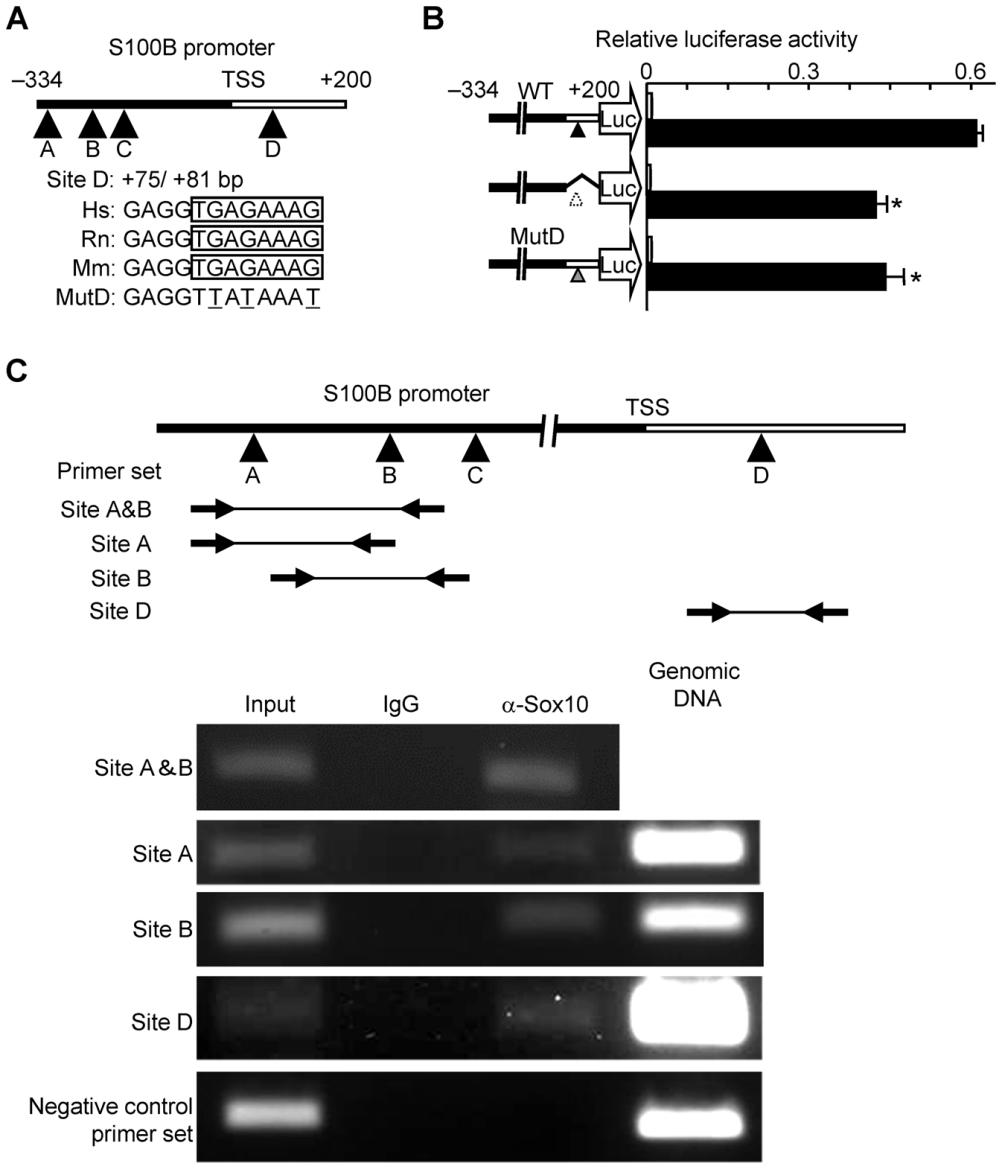
Identification of putative response elements in S100B intron 1 by SOX10 and direct binding of SOX10 to the response elements. (A) Comparison of human (Hs), rat (Rn) and mouse (Mm) sequences in the putative SOX motif of the S100B intron 1 and mutated sequence (Mut D), used in the following mutagenesis analysis. (B) Deletion and site-directed mutagenesis analysis using luciferase-reporter constructs containing −334 to +200 bp of the *S100B* gene in HeLa cells transfected with SOX10 or control GFP. *P<0.05 versus wild-type (WT) with SOX10. All experiments were repeated independently three times with data shown as the mean ± SEM. (C) ChIP assay performed using cell lysates of Schwann cells that were amplified by a primer set spanning the identified regions; sites A & B (top), site A (second row), site B (third row), and site D (fourth row), or not spanning the region (bottom) before (input) and after immunoprecipitation with antibodies to Sox10 (α-Sox10) or non-immune IgG (IgG). Genomic DNA was amplified as a positive control.

We then analyzed the promoter activity of human *S100B* using human HeLa cells transfected with a luciferase reporter gene construct containing a 5′-flanking sequence from −1,000 to +200 bp relative to the transcriptional start site of *S100B*. Among the transcription factors known to regulate Schwann cell differentiation, such as EGR2, POU3F, PAX3, and SOX10, SOX10 most potently enhanced transcriptional activity (Fig. 4A). Deletion analysis by a series of 5′-deletion constructs identified the location of the SOX10 response element between −334 and −240-bp (Fig. 4B). This region has been reported as a Sox10 binding site in ChIP-Seq analysis by Srinivasan et al. [27] and contains three putative consensus sites for SOX10, each of which was highly conserved among species like human, rat, and mouse (sites A, B, and C) (Fig. 4C). Site-directed mutagenesis of sites A and B but not of site C significantly suppressed transactivity of SOX10 (Fig. 4D). Deletion analysis using a 3′-deletion construct and site-directed mutagenesis demonstrated another putative SOX10-response element in the first intron (site D) (Fig. 5A–5B). A ChIP assay demonstrated the direct binding of SOX10 to the SOX motifs in sites A, B, and D (Fig. 5C).

### Contribution of SOX10-S100B signaling to proliferation and myelination of Schwann cells

**Figure 6:**
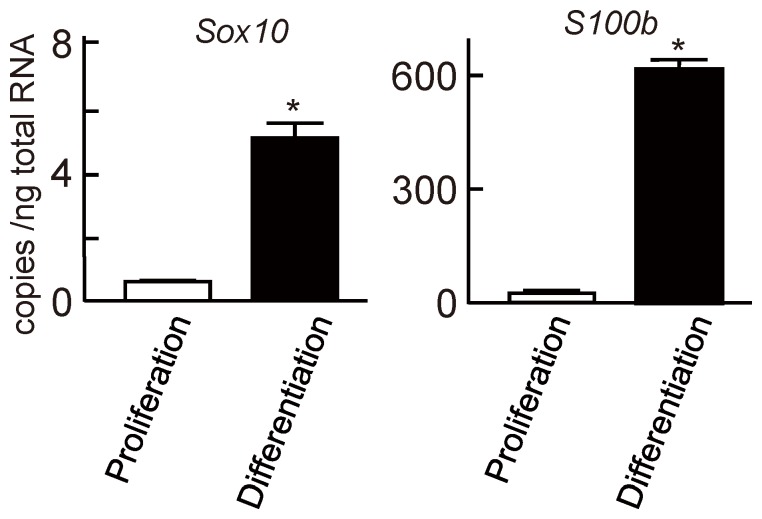
Suppressed Schwann cell proliferation by SOX10-S100B signaling. Comparison of *Sox10* and *S100b* mRNA levels between conditions of proliferation and differentiation in rat sciatic nerve Schwann cells. All experiments were repeated independently three times with data shown as the mean ± SEM. *P<0.05 versus proliferation condition.

**Figure 7:**
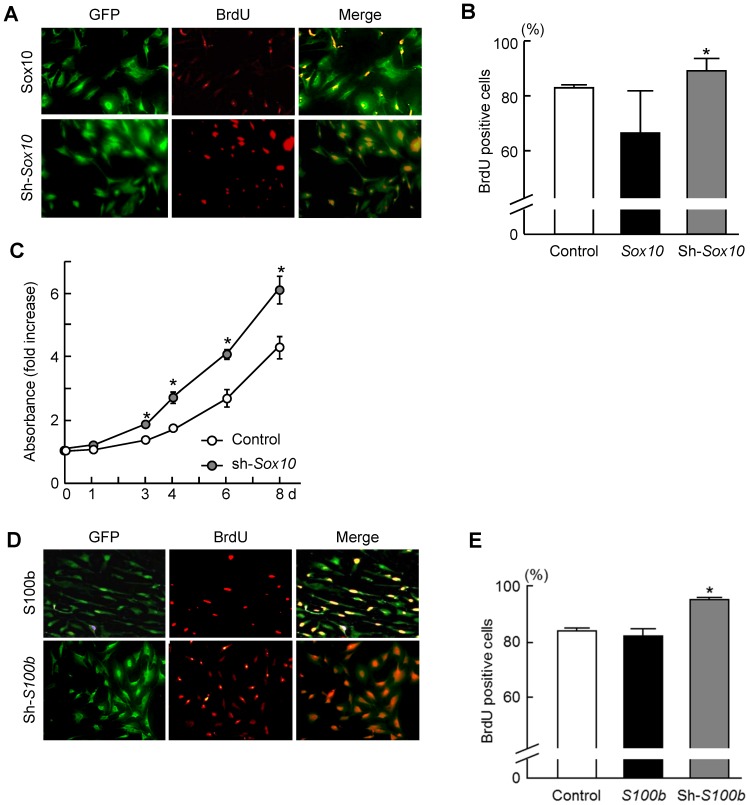
Enhanced proliferation by knockdown of *Sox10* or *S100b* in Schwann cells. (A, B) BrdU labeling of stable lines of Schwann cells retrovirally transfected with *SOX10* or shRNA specific for *SOX10* and *GFP* (A). Ratio of BrdU-positive cells to total cells was quantified after 3 d culture of stable lines of Schwann cells transfected with *Sox10* expressing vector, shRNA vector specific for *Sox10*, and control *GFP* vector (B). (C) Growth curves using the CCK-8 assay of stable lines of Schwann cells retrovirally transfected with sh-*Sox10* or control *GFP*. Experiments were repeated independently three times with data shown as the mean ± SEM. *P<0.05 versus control. (D, E) BrdU labeling of stable lines of Schwann cells retrovirally transfected with *S100b* or shRNA specific for *S100b* and *GFP* (D). Ratio of BrdU-positive cells to total cells were quantified after 3-day-old cultures of stable lines of Schwann cells were transfected with *S100b* expressing vector, shRNA vector specific for *S100b*, and control *GFP* vector (E). Experiments were repeated independently three times with data shown as the mean ± SEM. *P<0.05 versus control.

**Figure 8:**
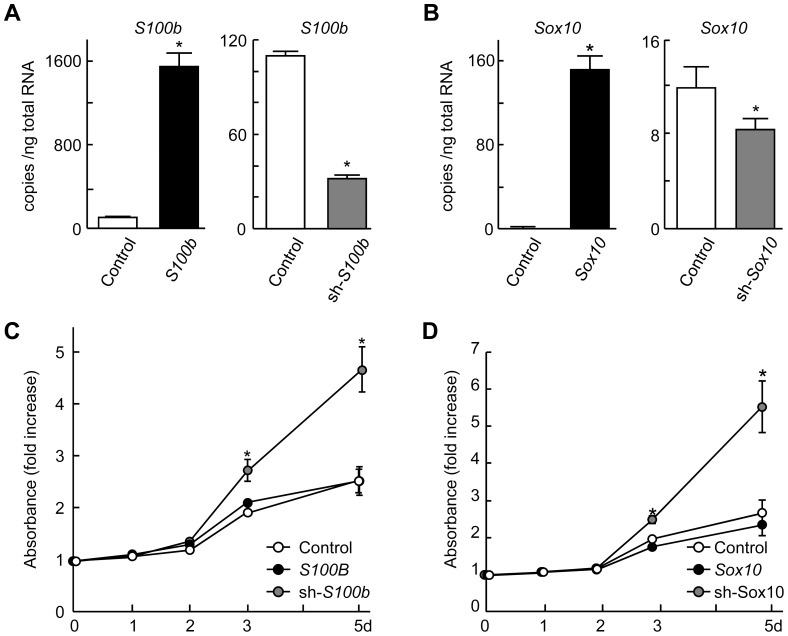
Enhanced proliferation by knockdown of *S100b* or *Sox10* in C3H10T1/2 cells. (A) mRNA levels of *S100b* determined by real-time RT-PCR in stable lines of mouse mesenchymal C3H10T1/2 cells retrovirally transfected with *S100b*, shRNA for *S100b*, or control *GFP*. (B) mRNA levels of *Sox10* determined by real-time RT-PCR in stable lines of mouse mesenchymal C3H10T1/2 cells retrovirally transfected with *Sox10*, shRNA for *Sox10*, or control *GFP*. (C and D) Growth curves using the CCK-8 assay of stable lines of C3H10T1/2 cells as mentioned above. All experiments were repeated independently three times with data shown as the mean ± SEM. *P<0.05 versus control.

We next examined the function of S100B in the proliferation of Schwann cells. When Schwann cells were cultured under the proliferation or differentiation conditions, i.e., with or without NGF and forskolin treatment, respectively [28], the expression of both *Sox10* and *S100b* was markedly suppressed under the proliferation condition but increased under the differentiation condition (Fig. 6). When we knocked down either *S100b* or *Sox10* with shRNA, BrdU incorporation significantly increased (Fig. 7A–B, 7D–E). In the CCK-8 assay, knocking down either *S100b* or *Sox10* in the Schwann cells or non-glial cells (C3H10T1/2) also increased cell proliferation (Fig. 7C and Fig. 8). These results suggest that SOX10-S100B signaling negatively regulates Schwann cell proliferation.

**Figure 9:**
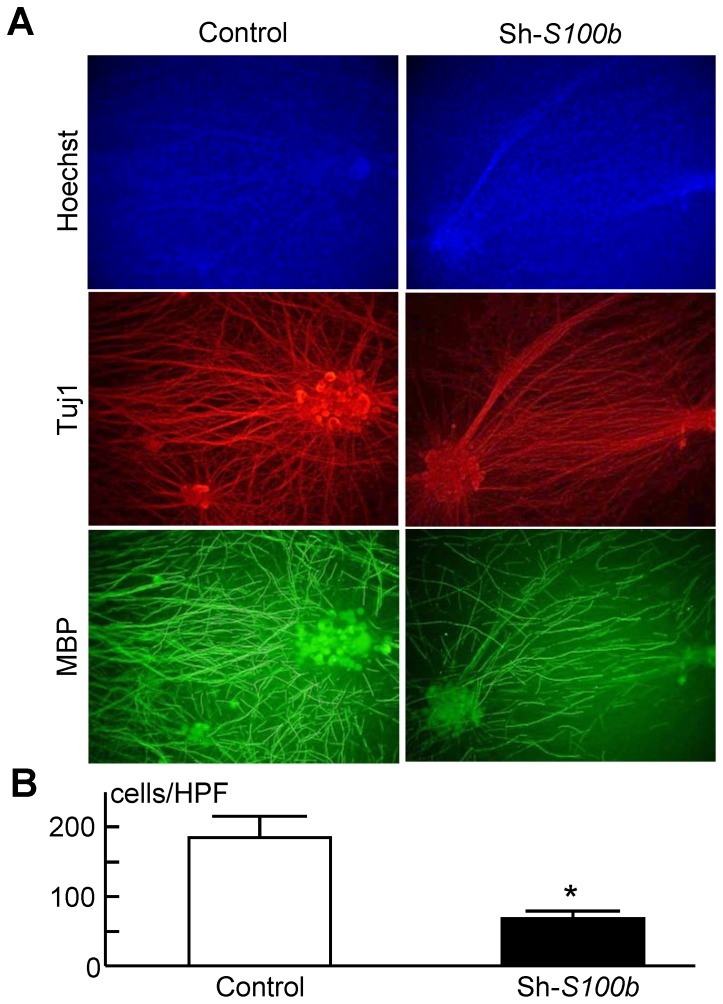
Impaired myelination by knockdown of *S100b*. (A) Immunocytochemistry of neurons and stable lines of Schwann cells retrovirally transfected with shRNA specific for *S100b* or control *GFP* in DRG dissociated cultures. Staining of Tuj1 (red), MBP (green) and Hoechst (blue) in neurons, Schwann cells, and nuclei, respectively. (B) The number of MBP-positive Schwann cells in a high-power field of the immunocytochemistry as in Fig. 9A. Experiments were repeated independently three times with data shown as the mean ± SEM. *P<0.05 versus control.

Finally, we examined the involvement of S100B in myelination using dissociated DRGs. Compared to control cocultures, knocking down *S100b* in Schwann cells impaired the myelination of rat DRG neurons (Fig. 9A), and we quantified this by calculating the number of MBP-positive myelinating cells (Fig. 9B). This result suggests that S100B in Schwann cells plays a critical role in myelination.

## Discussion

During vertebrate development, SOX10 is first highly expressed in the emerging neural crest and later in glial cells, where SOX10 is involved in the differentiation of both the peripheral nervous system (PNS) and CNS [7], [29]. SOX10 also plays an essential role in maturing and maintaining Schwann cells [30] by directly regulating MPZ, c-Ret, ciliary neurotrophic factor (CNTF), dopachrome tautomerase (DCT), microphthalmia-associated transcription factor (MITF), connexin32, connexin47, EGR2, SP1, and SP3 [31]–[39]. In the present study, we demonstrated that during Schwann cell differentiation, SOX10 is involved in the transcriptional induction of S100B. SOX10 belongs to the SOX transcription factor family that contains high-mobility group (HMG) domain(s) [40], [41]. The SoxE family contains SOX10, SOX9, and SOX8. The family members are structurally similar and are known to have functional redundancies [42], [43]. We previously reported that SOX9 regulates S100B expression in chondrocytes through direct binding to the *S100b* promoter(s) [17]. Here, we found that *Sox10* is predominantly expressed in Schwann cells as compared to *Sox9* (Fig. 1A), and the expression levels of SOX8 and SOX9 are lower than that of SOX10 in differentiated glial cells [36]. In addition, previous reports showed that the expression of S100B as well as MZP and MBP is suppressed in SOX10-deficient sciatic nerves [30]. Therefore, in the present study, we focused on the role of SOX10 in Schwann cell differentiation.

It has been reported that SOX transcription factors determine cell fate by enhancing transcriptional activity through interaction with their co-factors; several factors such as PAX3 [33], SP1, SP3, heterogeneous nuclear ribonucleoprotein K, pur-alpha [35], MITF [34], EGR2 [31], [36], and POU3F [44] have already been shown to function as co-factors of SOX10. In addition, because Sox10 has several known targets, such as Krox20/Egr2 and ErbB3, we could not exclude the possibility that these molecules also play a role in the regulation of proliferation and differentiation together with S100B. The cooperation of SOX10 with other factors should be analyzed to elucidate the mechanism of S100B induction in Schwann cells in further detail.

Although S100B is involved in energy metabolism, cell cycling, apoptosis, extracellular signaling, and regulating the cytoskeleton [11], [45], [46], its function in Schwann cells has not been fully clarified. We found that the expression of SOX10 and S100B was decreased under the proliferation condition, while it was increased under the differentiation condition (Fig. 6A). Moreover, knockdown of SOX10 or S100B increased the proliferation of Schwann cells (Figs. 6B, 7A–7B). A previous report showed a significant increase in the number of proliferating cells in the sciatic nerve from Schwann cell-specific *Sox10*-ablated mice [30]. By analyzing cyclin-dependent kinase (Cdk)-deficient mice, it was discovered that Cdk4 controls postnatal Schwann cell proliferation [47], although the exact role of SOX10 or S100B as a regulator of cell cycle-related molecules in Schwann cells has not been established. Interestingly, S100B promotes cell cycling in the CNS [9] and S100B levels are high in neuronal tumor cells as compared to normal parental cells. Nevertheless, other studies demonstrated that the Cdk inhibitor p21^WAF1^ was induced by S100B via AKT activation in PC12 neuronal cells [48] and that another Cdk inhibitor, p27^Kip1^, activated the MBP promoter in cooperation with SOX10 in oligodendrocytes [49]. Therefore, SOX10-S100B signaling may negatively regulate cell-cycle progression in Schwann cells by activating inhibitors of Cdks.

We also show that S100B is involved in Schwann cell myelination (Fig. 9). Our findings confirm a previous study that found that myelination is delayed in *S100b*-deficient mice [50]. How S100B functions to stimulate Schwann cell myelination is still unclear. Recent work suggests that because S100B is a Ca^2+^ binding protein, S100B controls intracellular Ca^2+^ concentration crucial for myelination induced by neuregulin-dependent phosphorylation of calcineurin. Neuregulin signaling controls myelination by increasing Ca^2+^ levels in Schwann cells in order to activate the phosphatase calcineurin [51], [52]. Thus, because Ca^2+^ levels regulate myelination, as a Ca^2+^ binding protein, S100B may also influence myelination in Schwann cells.

We conclude that SOX10 directly transactivates S100B to inhibit proliferation and to promote myelination during Schwann cell differentiation. It has been reported that the function of S100B changes depending on its expression levels. While at nanomolar levels S100B promotes axon extension via RAGE receptors, at millimolar levels S100B triggers apoptosis in neurons. Furthermore, S100B expression levels can indicate the malignant grade of malignant tumors [11], [53]. Together, these lines of evidence suggest that modulating the SOX10-S100B axis may be a viable therapeutic target for various neuronal disorders including demyelinating disease, neuropathy, and nerve injury.

## Acknowledgments

We thank R. Yamaguchi and H. Kawahara for excellent technical assistance.
